# From Feline Idiopathic Ulcerative Dermatitis to Feline Behavioral Ulcerative Dermatitis: Grooming Repetitive Behaviors Indicators of Poor Welfare in Cats

**DOI:** 10.3389/fvets.2018.00081

**Published:** 2018-04-16

**Authors:** Emmanuelle Titeux, Caroline Gilbert, Amaury Briand, Noëlle Cochet-Faivre

**Affiliations:** ^1^Ecole Nationale Vétérinaire d’Alfort, Unité Ethologie, Maisons-Alfort, France; ^2^UMR 7179, CNRS/MNHN, laboratoire MECADEV, Brunoy, France; ^3^Ecole Nationale Vétérinaire d’Alfort, Unité Dermatologie, CHUVA, Maisons-Alfort, France; ^4^Advetia, Paris, France; ^5^Clinique vétérinaire des Halles, Strasbourg, France

**Keywords:** cat, feline, head and neck pruritus, idiopathic ulcerative dermatitis, repetitive behavior, welfare

## Abstract

Feline idiopathic head-and-neck dermatitis—also named feline idiopathic ulcerative dermatitis (IUD)—is considered as a rare skin disease of unknown origin. It is usually associated with a crusted, non-healing, self-induced ulcer occurring most commonly on the dorsal or lateral neck or between the scapula where self-grooming by scratching occurs. Usually, IUD is diagnosed after exclusion of other causes of pruritus. In feline medicine, self-induced alopecia is recognized as a behavioral disorder (abnormal repetitive behavior) due to excessive licking, which is an amplification of a normal maintenance behavior. Such repetitive behaviors, like self-induced alopecia or self-induced wounds, are named stereotypies and considered as indicators of poor welfare. The objectives of our study were to determine, first, if the repetitive behavior associated with self-induced wounds was related to a poor welfare, and, second, if improving the welfare in the cat’s environment would lead to healing, thanks to environmental enrichment. We recruited 13 cats diagnosed with IUD by a dermatologist. These cats were referred to a behaviorist for welfare evaluation. A welfare score was attributed using a new 21-point welfare scale. The median score of the 13 IUD cats was 16, while the median score of 35 healthy cats was 7 (significant difference, *p* < 0.001). Major modifications of the cat’s environment and the human–cat relationship were then recommended for IUD cats. Within 15 days after environment modifications, ulcerative lesions were healed and welfare scores improved significantly (median score of 6, significantly different from the score before environmental modifications), being similar to healthy cats (no significant differences). Only one cat was treated with a psychotropic drug, owners being reluctant to improve environmental modifications. These results suggest that feline IUD is a behavioral disorder indicative of poor welfare and that it requires management by behavior specialists, proposing environmental modifications. We hence propose to rename this affection to “behavioral ulcerative dermatitis,” given that welfare scores were significantly different from healthy cats, and that environmental modifications modified welfare scores and lead to successful healing in all cases.

## Introduction

In cats, head and neck pruritus (HNP) is a dermatologic syndrome consisting of pruritus located to the head and/or the neck combined with cutaneous lesions. Most of the time, cutaneous lesions are excoriations, i.e., self-induced erosions or ulcerations and can worsen primary cutaneous inflammatory lesions such as miliary dermatitis, eosinophilic plaque, or urticarial papules. Excoriations can be initiated by a pruritic sensation without any inflammatory skin condition or can be a consequence of an underlying pruriginous dermatosis. Causes of HNP are multiple but in some cases, cannot be identified, which lead to a diagnosis of feline idiopathic HNP also named feline idiopathic ulcerative dermatitis (IUD).

Idiopathic ulcerative dermatitis is considered as a rare skin disease of unknown origin that was first reported in 1990 (1). It is characterized by self-induced lesions generally located around the neck, on the temporal areas, or between scapulae of cats. Clinically, lesions are erosive or ulcerative with sometimes deep ulcers surrounded by a border of thickened skin (2, 3). Lesional patterns can be symmetric or asymmetric. A peripheral lymphadenomegaly may be present due to inflammation and/or secondary infection (2). No signs of systemic illness are present. Classical differentials include, according to the lesion area, foreign body reaction, trauma, thermal burn, erythema multiforme, bacterial, fungal or viral infection, parasitic infestation, hypersensitivity disorders, neuropathic disorder, and neoplasia (2–6). Histopathological examination describes extensive epidermal ulceration and superficial dermal necrosis with minimal to mild dermal inflammation composed in majority of neutrophils, few mononuclear cells, and rare eosinophils. Chronic lesions may also have a subepidermal band of dermal fibrosis extending peripherally from the ulcer (7). Ulcerative dermatitis heals spontaneously as soon as the cat is prevented from self-mutilation with coercive measures, like e-collar or bandages. This disease is refractory to the majority of medications, except corticosteroids for a short period, before relapse is observed. Successful treatment is anecdotally described with topiramate, gabapentine, cyclosporine, or oclacitinib (6, 8). Wide surgical excision may be attempted but is often unsuccessful. The prognosis is, therefore, guarded and relapse may occur rapidly after the removal of protective bandages or systemic treatment. Currently, its aetiopathogenesis is not understood and the underlying cause has not been determined yet. Hence, diagnosis of feline IUD is still a diagnosis of exclusion and an effective treatment has not been identified yet.

Nevertheless, another cat grooming disorder, the self-induced alopecia, is now considered as psychogenic (9, 10) and even named “stress-related overgrooming” (11). Environmental stress has been detected and treatment associating environment modifications (12, 13) and antidepressant drugs are being proposed (14). In various animal species, abnormal repetitive behaviors (ARBs), named stereotypies for some authors, are considered as indicators of welfare problems (15, 16). They are also used as indicators for poor welfare scores when present (16–18). Overgrooming is currently described as an indicator of poor welfare especially in species whose allocate a significant part of time-budget to grooming (13, 19). In chinchillas, for example, fur chewing is classified as ARB and is linked with inappropriate and restrictive environmental conditions (19). Grooming represents 4% of the daily activity budget of cats, i.e., 8% of non-sleeping or resting time (20), which is a high proportion of their time-budget. Grooming behavior by cats is expressed in three different ways through licking, biting, and scratching (21). Scratching behavior represents 1–2% of time devoted to grooming (21) and is performed with the hind paw and claws half out. Scratching is limited to the neck, the cheek, under, and behind the ears (21). If prevented from scratching itself for several days, a cat will show an increase of 200% in the amount of scratching during the first 12 h it is permitted (20, 21). In cats, the control of grooming behavior is supposed to be central (20).

Current accepted definitions of welfare are based on a multidimensional concept, defined as a state of complete mental and physical health where the animal is in harmony with its environment experiencing positive emotions (22), its adaptations being successful and easily implemented with a minimum of stress reactions (23, 24). Considering the evaluation of welfare, specialists have designed animal-based measures for the overall welfare assessment of cattle, pig, and poultry [Welfare Quality^®^, (25)]. Welfare quality^®^ assessment identifies four principles: good feeding, good housing, appropriate behavior, good health; and twelve criteria: absence of prolonged hunger, absence of prolonged thirst; comfort around resting; thermal comfort; ease of movement; absence of injuries; absence of disease; absence of pain induced by management procedures; expression of social behaviors; good human–animal relationship, and absence of general fear [(25), Animal Welfare INdicators AWIN developed for several animal species]. For example, for horses, indicators (26) have been developed using the concept of welfare quality^®^, with the item “presence of stereotypy” taken into account and considered as an indicator of poor welfare.

However, considering dogs and cats, no welfare scores such as Welfare quality^®^ or AWIN have been proposed yet. Developing an adapted welfare score could be an interesting tool to assess the welfare of cats suffering from IUD. Considering the importance of grooming in cat activity budget and the well-recognized stress-related overgrooming linked to environmental factors, we hypothesize that IUD could be a consequence of environmental factors and a sign of a poor welfare as well. It has also been proven that environmental enrichment (i.e., addition of objects or stimulations in the environment of an animal to diversify its behaviors and improve its welfare) reduces stereotypies (12, 13). The objective of the study reported here was to show that IUD is associated with behavior disorders linked to environmental factors. Our first hypothesis was that cats suffering from IUD would present welfare scores significantly different from healthy cats and our second hypothesis was that an enrichment of the environment improving welfare scores would lead to a cure of the disease.

## Materials and Methods

### Study Design

The study was designed as a prospective open controlled study. All cats were recruited from January 2014 to January 2016 at Alfort School Veterinary Hospital, France (CHUVA). Cats were treated according to the CHUVA ethics, since they were recruited as patients from dermatology consultations or vaccine consultations for control cats. IUD cats were under care at the CHUVA, and owners of control cats were asked to fill out a questionnaire. All owners gave their written consent to participate to the study.

### Welfare Score

**Figure 1:**
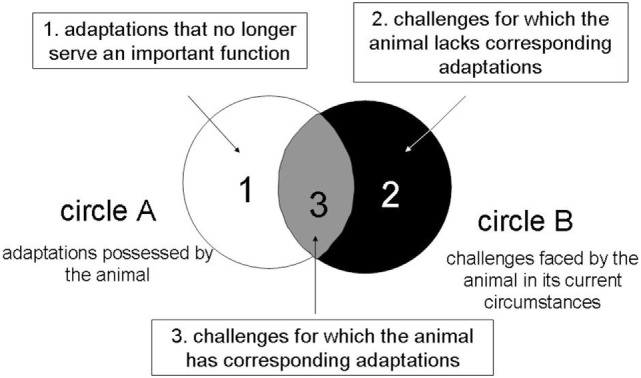
Conceptual model illustrating problems that may arise when the adaptations possessed by the animal (circle A) make an imperfect fit to the challenges it faces in the circumstances in which it is kept (circle B) (with D. Fraser permission).

A welfare score (Table 1) was built based on welfare scores developed on cattle, pig, poultry and horses [Welfare Quality^®^ (27) and AWIN scores https://air.unimi.it/retrieve/handle/2434/269097/384836/AWINProtocolHorses.pdf]. The cat welfare score developed in this study incorporated previous concepts of welfare assessment and assessed the controllability of cats over their resources and environment. In particular, through a detailed questionnaire to owners, we assessed whether cats could freely access to food or water, to hiding or exploration spaces. “Cat controlled” means that cats had a full access to resources or spaces whenever they wanted, “partly owner controlled” means that cats had access to resources or spaces when asking to owners (i.e., by vocalizations), “totally owner controlled” means that cats did not have access to resources or space, which were controlled by owners (e.g., a cat asking to go outside, with an owner who did not open the door). We also investigated whether the relationship between owners and cats was negative or positive (i.e., whether interactions were only initiated by owners, or by the cat and owners, or by the cat only), and between the patient and other cats if any. The quality of environmental enrichment was assessed: whether objects the cat could use to play, to explore, were present or not. The adequacy between genetic and individual needs and environment was also estimated according to Fraser et al. (24) concept (Figure 1). This model gathers previous conceptions of animal welfare in order to conceptualize the challenges an animal has to face in its own environment. This concept is fitted to the behavioral consultation because it integrates subjective experience and thus the evaluation of welfare may be implemented for one animal in its own environment.

**Table 1:** Welfare score used in the study.

Indicators	Score		Measures; questions to owners	Signification
Medical exam: presence of wounds	No	0	Clinical exam: presence or absence of wounds	Presence or absence of pain
	Yes	1		

(I) Adequacy between the cat’s genetic needs and its living conditions	No	1	Does your cat come from the countryside? Did he/she show signs of fear or anxiety during the first months? Did your cat spend time hidden?	Inadequacy between genetic and environmental conditions (24)
	Yes	0		
(II) Access to food and water resources				Investigate any frustration for the cat to access to food and water

(a) Food	Cat controlled	0	How much and how food is available? Does your cat meow to get food?	
	Partly owner controlled	1		
	Totally owner controlled	2		

(b) Water	Cat controlled	0	Is clear water easy to find? Does your cat ask for running water from the tap?	
	Totally owner controlled	1		

(III) Access to space (hide and explore)				Investigate any frustration for the cat to access to hiding places, to rooms, to exploration sites (window, balcony, outside)

(a) Rooms and hiding places	Cat controlled	0	Does your cat have a free access to each part of the apartment/house? Has your cat hiding places? Is the cat allowed to sleep in closets?	
	Partly owner controlled	1		
	Totally owner controlled	2		

(b) Windows	Cat controlled	0	Does your cat have a free access to windows? Do you accept if your cat asks to spend time on the edge of window? When the weather is mild, do you accept to let a window opened all day long?	
	Partly owner controlled	1		
	Totally owner controlled	2		

(c) Balcony	Cat controlled	0	Is your cat allowed to spend a day long on the balcony? Is the balcony opened as soon as your cat meows in front of the door?	
	Partly owner controlled	1		
	Totally owner controlled or no balcony	2		

(d) Outside	Cat controlled	0	If you have a garden, is your cat allowed to get out? Do you have a catdoor? If not, do you open the door as soon as your cat asks for the opening?	
	Partly owner controlled	1		
	Totally owner controlled or no garden	2		

(IV) Relationship between cat and owners	Cat controlled	0	Do you carry and hold your cat in your arms? Do you stroke your cat when you want? How does your cat react when you pet him/her? How does your cat react if he/she sleeps on the sofa and you come just for seating?	Investigate the quality of the relationship between the cat and owners
	Partly owner controlled	1		
	Totally owner controlled	2		

(V) Relationship between cat and other cats (in case of multiple cat household)	Affiliative tolerance or no cats	0	Do you observe allogrooming, allorubing between your cats? Do you observe your cats sleeping together? Does your cat sleep near to other cats? Do your cats fight? How does your cat react when he/she is sleeping and another cat enters the room or decides to take its place?	Investigate the quality of the relationship between cats
	Partly mixed of agonistic and affiliative	1		
	Solely agonistic	2		

(VI) Cat activity-budget/enrichment/diversification of activities	Plays frequently (more than 1 h/day)	0	Could you tell us what is a typical day like for your cat? Are some toys (catnip, fishing rod toys, wire-base toys, balls…) present in your house?	Investigate the activity budget, the more diverse the activities are (and enrichment), the better
	Plays from time to time (less than 1 h/day)	1		
	No toy, no play	2		

(VII) Adequacy between the cat’s temperament and the environment	Full adequacy	0	Is your cat shy, bold, fearful, prone to attack, playful, familiar to humans? During the exam is the cat at ease, prone to play, prone to explore, prone to interact with us?	Inadequacy between temperament and environmental conditions (24)
	Mitigated	1		
	No adequacy	2		

Total				
Minimum score = “good” welfare score		0		No problem of welfare
Maximum score = “poor” welfare score		21		Serious welfare problem

The welfare score notation system was numeric: for each question two or three propositions were presented to the owners, scored 0, 1, or 2 according to the number of propositions. For each question, the answer scored 0 represented the best option (according to welfare criteria) whereas a 1 or 2 scored answer represented worst options. The global welfare notation was the sum of all the scores obtained from the questionnaire: the maximum total score was 21 and would correspond to serious welfare problem whereas a total score of 0 would show the absence of welfare problem.

### Animals

#### Healthy Cats

Cats were recruited from the preventive veterinary medicine service from January 2014 to January 2016. Animals were recruited during a vaccination consultation. Cats younger than 6 months old or suffering from a chronic disease, a dermatologic condition or a known behavior disorder were excluded. Cat owners were then given a questionnaire referring to the Welfare score newly developed. Each cat was then given a global welfare score.

#### IUD Cats

Cats suffering from IUD were recruited from the dermatology service from January 2014 to January 2016 and were assessed by a dermatology specialist (ECVD diplomate). The diagnosis was based on characteristic clinical features, i.e., self-induced lesions or excoriations in the region of the head and neck (which correspond to normal areas of grooming by scratching) and exclusion of other pruritic dermatoses. Other pruritic conditions included in the differential diagnosis, such as atopic dermatitis, cutaneous adverse food reaction, flea-allergy dermatitis, external parasitism, bacterial or fungal dermatitis, metabolic conditions, or other inflammatory dermatoses, were eliminated through thorough physical and dermatological examination and, if necessary, appropriate complementary exams. All cats recruited had received regular flea-control for at least 3 months before entering the study. Concomitant administration of drugs was not an exclusion criterion. After dermatological assessment and a diagnosis of IUD was made, cats were referred to a behavioral specialist. The aim of the behavioral consultation was to explore the adequacy between behavioral needs of cats and their environment, in order to assess whether they would be or not in poor welfare conditions using the newly developed welfare score. A first welfare score was calculated the day of the first consultation at inclusion (S1) and then a second welfare score was given during the next recheck (S2), the interval of time between S1 and S2 varying from 15 to 90 days.

### Behavioral Treatment: Environmental Enrichment

The behavioral consultation lasted approximately 1 h and a half. Genetic origin, behavioral development, cat’s temperament, access to resources (food, water, litter box, hiding places, resting places), time-budget, human–cat relationship, inter-cat relationship, and enrichment were carefully assessed in order to fill in the welfare score. The anamnesis consisted in listing each situation possibly impairing the cat welfare: distress, conflicts, and frustrations. Following our hypothesis, IUD being the result of the discrepancy between the cat ethological needs and its living conditions, the modification of its environment should obviously lead to resolution of the condition. The living conditions modifications implemented in our study followed the usual recommendations proposed in previous publications (28–30). Environmental enrichment can be defined as “any addition to the environment of an animal resulting in a presumed increase in the environment’s quality, and a subsequent presumed improvement to the animal’s welfare” (31). Animate and inanimate strategies were then proposed to the cat in order to (28, 29):

- Increase behavioral diversity;
- Reduce the frequency of abnormal behavior;
- Increase the range or number of “normal” (i.e., species-typical) behavior patterns;
- Increase positive utilization of the environment;
- Increase the ability to cope with challenges in a more “normal” way.

We, therefore, gave several recommendations for each IUD cat, adapted to each case.

#### Removing All Frustrations and Restoring Control on Its Environment

Food access should be permanent. For cats with excessive food intake a low-calorie food was proposed and presented in a Trixie fun board^®^, which increased time devoted to foraging. For some cats, a water fountain was proposed because during the consultation the owner mentioned that their cat “asked” to drink running water from the tap and drank rarely in a bowl.

For exercise and exploration, the opportunity for the cat to have a free access to garden or balcony or window was proposed to the owner. Examples of balconies or windows designed and secured for cats were shown to owner during the consultation. The installation of a cat door was firmly recommended.

#### Improving the Cat–Human Relationship

We recommended stopping the interactions initiated by owners (carrying the cat, petting the cat). We explained to the owner that interactions should hence be initiated by the cat. Owners were advised to reinforce interactions positively with treats.

#### Changing Cat–Cat Relationship in Case of Multiple Cat Household

Owners were advised to provide access to secure area where each cat could eat, sleep, and eliminate urine and feces without being in competition with one another. In some situation, it could be advised to perform a complete separation of the cat life area using the different floor of the house when possible and during few weeks.

#### Adaptation of Time-Budget and Environment to Cats’ Needs

Owners were advised to regularly offer new toys to its cat (a large range of toys was proposed during the consultation) and should propose high and hidden resting areas. Closets should be let opened if the cat uses it as hideout for instance.

### Medical Treatment

No medical treatment was prescribed during the study period. Environmental changes were the only interventions. All treatments prescribed between the dermatological and the behavioral consultation were stopped after the behavioral consultation (Table 2). In one case (cat 13), owners could not make any environmental modification and the cat received medical treatment instead: fluoxetine (fluoxetine 1 mg/kg Sandoz, France; daily) for 1 month and then imepitoin (Pexion Boehringer-Ingelheim, Germany, 10 mg/kg; twice daily) for another month.

**Table 2:** Identification, clinical presentation, previous analyses, and treatments related to the idiopathic ulcerative dermatitis cats.

Cat	Breed	Sex	Age of onset (months)	Duration of disease before presentation	Localization	Previous exams	Previous treatment before dermatology consultation	Treatment between dermatological and behavioral consultation
1	DLH	FN	20	1 year	Temporal areas, lateral neck	FIV FeLV, video otoscopy scanner cytological exam	FT, MPA, MP, CsA, AB, AH, RDiet, Fluoxetine	None

2	Maine coon	M	9	1 month	Dorsal neck	Cytological exam	FT, RDiet	None

3	DSH	MN	24	2 years (flare ups)	Lateral neck	FIV FeLV, histological exam, cytological exam	FT, MPA, RDiet	None

4	DSH	MN	Unknown	Unknown	Dorsal, lateral neck	FIV FeLV, fungal culture, cytological exam	FT + unknown	None

5	DSH	MN	24	2 years (flare-ups)	Bilateral lateral neck	FIV FeLV, histopathological exam	FT, MP, MPA, AH	Cetirizine, Gabapentin

6	Maine coon	FN	18	1 year (2 flare-ups)	Dorsal neck	Fungal culture	FT, MPA	None

7	British shorthair	MN	18	9 months	Lateral neck	FIV FeLV, histopathological exam, bacteriological exam	FT, MPA, AB, Oclacitinib, CsA, AH, Gabapentin, RDiet	Gabapentin, MP, CsA, Cetirizine

8	Scottish fold	FN	6	8 years (2 flare ups/year) the last > 6 months	Ventral neck, chin	Fungal culture, cytological exam	FT, MPA, RDiet	None

9	DSH	FN	40	3 months	Dorsal neck	FIV FeLV, cytological exam	FT, MP, AH, Selegiline	Gabapentin

10	DSH	MN	36	2 months	Unilateral temporal area	Fungal culture	FT, MP, RDiet	Gabapentin

11	Maine coon	FN	36	2 years	Unilateral shoulder blade	Histological exam, CBC, biochemical profile	FT, MP, RDiet	Gabapentin

12	DSH	FN	16	9 months	Unilateral retroauricular area	Fungal culture	FT, MPA, RDiet	Gabapentin

13	DSH	FN	12	5 months	Unilateral lateral neck	Cytological exam	FT, MPA, CsA, AB, AH, Oclacitinib, Fluoxetine	None

*AB, antibiotic; AH, antihistaminic; CBC, complete blood count; CsA, ciclosporin A; DLH, domestic long hair; DSH, domestic short hair; FeLV, feline leukemia virus; FIV, feline immunodeficiency virus; FN, female neutered; FT, flea treatment; MP, methylprednisolone; MPA, methylprednisolone acetate; MN, male neutered; RDiet, restriction diet*.

### Statistical Analyses

In order to compare welfare scores of IUD and healthy cats, we performed Mann–Whitney tests for non-parametric distribution and to compare S1 and S2 for IUD cats, Wilcoxon tests were performed. Significance was determined at *p* < 0.05.

## Results

### Animals

#### Healthy Cats

Thirty-five healthy cats were recruited during this study. They were all domestic short hair, except one Chartreux and one Blue Russian. Age of cats varied between 1 year and 16 years old (median age was of 7 years), 15 were castrated males and 20 neutered females. 6 cats had a free access outdoor, 19 a controlled access, and 10 cats had no access.

#### IUD Cats

**Figure 2:**
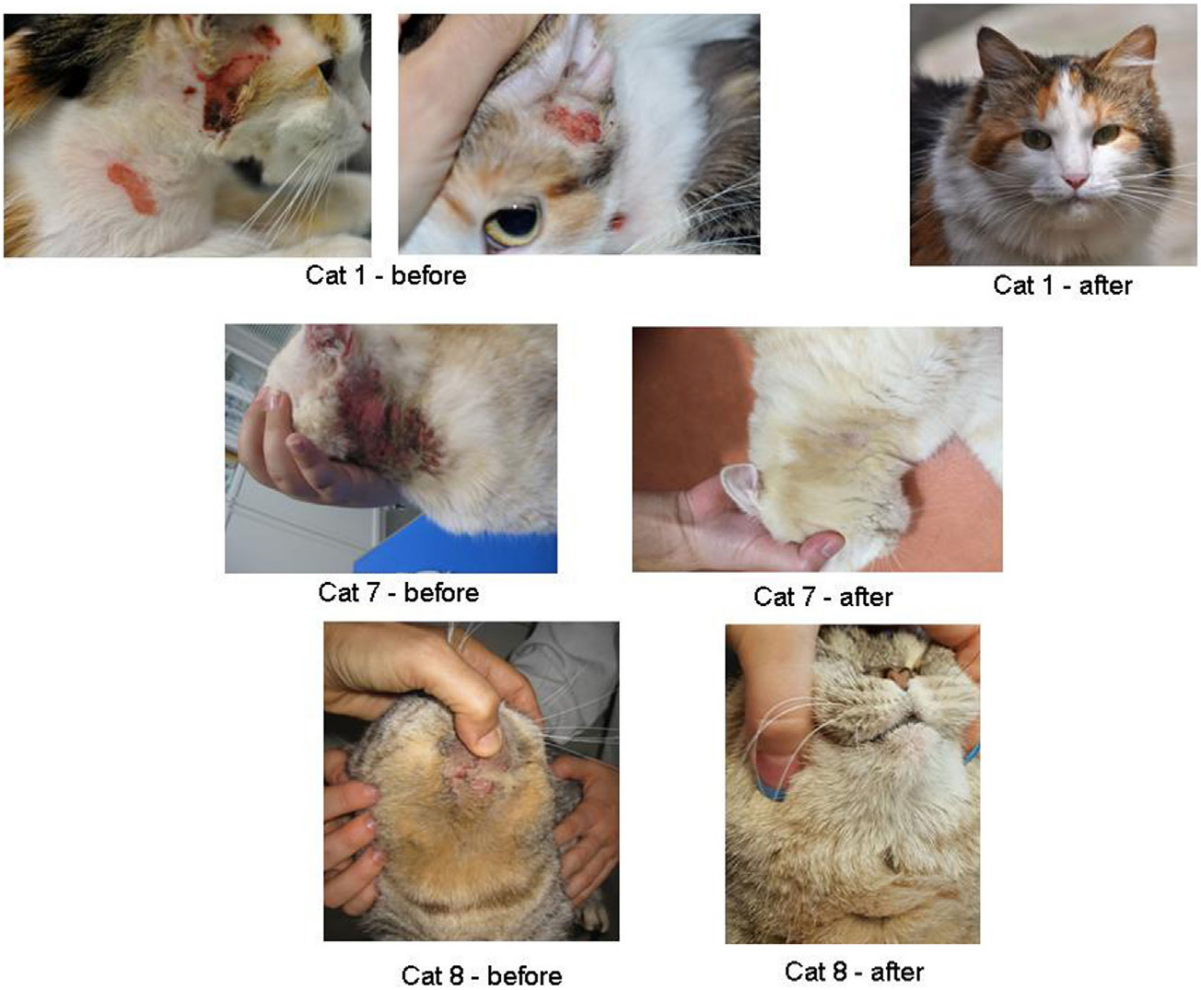
Photos illustrating dermatological aspect before and after environmental modifications (with owner’s permission).

Thirteen cats were recruited in this group (Table 2; Figure 2). Except cat 2, all cats were referred by generalist vet to dermatologist specialist because of failure of treatment. This group was composed of 6 males (5 castrated) and 7 neutered females. Age of cats varied between 10 months old and 8.5 years old (median of 31 months). One cat was of unknown age. Of the 13 cats, 7 cats were Domestic shorthair cats. Other breeds were: Maine Coon (*n* = 3), British Shorthair (*n* = 1), and Scottish fold (*n* = 1).

The majority of cats lived strictly indoor (*n* = 11), the other two cats lived both indoor and outdoor but could undergo temporary indoor confinement.

Four cats have had a histopathological exam compatible with results observed in IUD. Eleven cats have had corticotherapy (Table 2). Nine owners have had reported a transitory improvement. No improvement at all was reported in two cases (Cases 12 and 13). For cat 4, previous treatments were unknown.

### Dermatological Examination

Age at onset of IUD varied between 6 and 40 months (median: 19 months) and duration of disease before presentation at the CHUVA consultation varied between 1 month and 8 years (median: 9 months). In all cats, the predominant clinical signs were excoriations (Table 2).

Localization of lesions were variable but always concerned the head (*n* = 4), the shoulder (*n* = 1), and/or the neck (*n* = 10). In two cats, the cutaneous lesions affected the head only, more precisely, the retroauricular (*n* = 1) or temporal area (*n* = 1).

The majority of cats presented cervical lesions (*n* = 10). Among them, two cats presented cervical lesions associated with other localization such as the chin (*n* = 1), or the temporal area (*n* = 1).

The majority of cats presented only one localized lesion (*n* = 8), the others presented two cutaneous lesion (*n* = 1) or more (*n* = 4).

### Results of Welfare Scores and Responses to Therapies

Healthy cats had significantly lower scores (i.e., revealing better welfare conditions) than IUD cats at inclusion (S1) [healthy cats: 7 (5; 8); IUD cats: IUD cats at S1: 16 (14.5; 17.5); *U* = 542.5, *p* < 0.001; Table 3]. Interestingly, when cats were cured with environmental modifications, their scores were significantly reduced [IUD cats at S2: 6 (4.5; 8); *W* = −91, *p* < 0.001; Table 3]. Moreover, when we compared the S2 score of IUD cats with healthy cats score, no differences were observed (*U* = 281, *p* = 0.387).

**Table 3:** Scores of idiopathic ulcerative dermatitis cats before (S1) and after (S2) behavioral consultation.

Cats	Wounds	I.	II.a	II.b	III.A	III.B	III.C	III.D	IV.	V.	VI.	VII.	Result
**S1 scoring**													
1	1	1	2	1	2	2	2	2	2	0	2	2	19
2	1	0	2	1	2	2	2	2	1	1	2	2	18
3	1	1	1	0	1	2	2	2	0	0	1	2	13
4	1	1	0	0	2	2	2	2	2	2	2	1	17
5	1	0	0	1	0	2	2	2	1	2	0	1	12
6	1	0	0	1	2	1	2	2	2	1	2	2	16
7	1	0	2	0	0	2	2	2	2	0	2	2	15
8	1	0	0	1	0	2	2	2	2	0	2	2	14
9	1	1	2	0	2	1	2	2	2	1	1	2	17
10	1	0	2	0	2	2	2	2	0	2	1	2	16
11	1	0	2	0	2	2	2	2	1	1	1	2	16
12	1	1	2	1	2	0	2	2	0	2	1	1	15
13	1	1	2	0	2	2	2	2	2	0	2	2	18

**S2 scoring**													
1	0	1	2	1	0	0	0	0	0	0	0	0	4
2	0	0	1	1	0	2	2	2	0	1	0	1	10
3	0	1	0	0	0	0	2	2	0	0	0	0	5
4	0	1	0	0	0	0	0	0	1	2	2	1	7
5	0	0	0	0	0	2	2	2	0	0	0	0	6
6	0	0	0	1	2	1	0	0	0	1	1	0	6
7	0	0	0	0	0	0	2	2	0	0	0	2	6
8	0	0	0	0	0	0	1	2	0	0	0	0	3
9	0	1	0	0	0	1	2	2	0	1	1	1	9
10	0	0	0	0	1	0	0	0	0	0	1	1	3
11	0	0	0	0	1	0	0	2	1	1	1	1	7
12	0	1	0	0	0	0	0	0	0	2	1	1	5
13	1	1	0	0	2	2	2	2	2	0	2	2	16

Considering IUD cats, as soon as environmental changes were set up, pruritus stopped within 2 days in all cats. Consecutively, skin lesions healed quickly thereafter in the following days (with or without scars depending on the wound depth).

All cats, except one (cat 13) healed. Indeed, for this cat, owners did not change the environment; hence an unsuccessful medical treatment was given. For 12 out of 13 cats with IUD (92%), clinical signs did not relapse during a follow-up varying between 12 and 24 months.

## Discussion

As in humans, psychogenic pruritus in cats is often mislabeled as idiopathic pruritus because we had so far, no other diagnosis to propose (32). A too rapid misdiagnosis may have severe consequences in the medical and welfare management of the cat as well as financial and psychological impact for the owner.

Although not lethal, IUD can deeply impact the human–cat relationship. Besides, the impact on both the owners and the animal quality of life can lead to euthanasia.

### IUD As Abnormal Repetitive Behaviors Linked With Poor Welfare Conditions

The organization and the regulation of grooming in cats is thought to be under the control of a central mechanism (20). In other species, it has been shown that this control could be deregulated by poor welfare (19) and leads to abnormal repetitive behaviors like fur chewing (19). In internal medicine, it is now well recognized that stress induced by environment has a strong influence in the aetiopathogenesis of idiopathic cystitis (33). A multimodal environmental modification provides a clear improvement of low urinary tract signs in cats with idiopathic cystitis (34). The term “Pandora syndrome” is proposed to describe cats with chronic recurrent low urinary tract signs in the presence of comorbid disorders (behavioral, dermatological, endocrine, gastrointestinal tractus) (35). Thus, the link between stress and aetiopathogenesis of multiple disorders in cats has already been suggested. This study extends the concept to a measured poor welfare.

In that context, we developed a cat welfare score based on AWIN criteria to assess whether IUD could be linked to poor welfare conditions and hence triggered by a problem of adaptation to the cat’s environment.

Interestingly, the 13 IUD cats diagnosed with IUD had a score related to a significantly poorer welfare condition (median score of 16, related to poor welfare) compared with healthy cats (median score of 7, significantly different). Through environmental modifications proposed during a behavioral medicine consultation, the score of IUD cats significantly decreased (to a median of 6, related to better welfare conditions) and were not significantly different from healthy cats. Interestingly, concomitantly to environmental conditions, IUD cats all healed quickly in the following days. All owners followed our recommendations except one owner (cat 13) who could not follow our prescription. This cat did not heal, and we had to prescribe psychotropic drugs without success.

So far, as all modifications for improving welfare were recommended to be straight implemented, it is difficult to assess which component had more influence on healing. However, for one cat (no. 6), the owner tried unsuccessfully to improve the environment without free access to outdoor. As soon as the cat had the opportunity to get out, he healed rapidly. Free access to outdoor (for instance with cat door installation) seems to be the trigger of an insured healing for many cats. Effectively, when a cat has a free access to outdoor it results in 10 points drop of the welfare score: 2 for access to window, 2 for access to balcony, 2 for access to outdoor, 2 for enrichment, and 2 for adequacy between cat and its environment.

### From “Idiopathic” to “Behavioral” Ulcerative Dermatitis

Because of the limited number of cases, we cannot conclude that all idiopathic HNP in cats are relevant for behavioral medicine, but we hypothesize that most of these cases are a manifestation of poor welfare conditions and abnormal repetitive behaviors. It appears important to us to rename this disorder. As IUD cats heal after environmental modifications associated to welfare improvement, we hence propose the term of behavioral ulcerative dermatitis or self-induced ulcerative dermatitis (by similarity to self-induced alopecia). The prognosis is good if the owner accepts and has the ability to perform the modifications of the cat living conditions.

For some cats, which lived with another cat, the other cat seemed to be healthy and seemed to be adapted to their environment. This is consistent with the conceptual model of Fraser et al. (24), which describes that some individuals are more able to cope with poor living conditions. Our results are also compatible with a genetic component of repetitive behaviors (36–38). Despite our small sample, breed cats were over represented compared to the general cat population [4.2% breed cats in the general population; (39)], suggesting a genetic predisposition to environmental mal-adaptation.

### Behavioral Ulcerative Dermatitis: Not a Diagnosis by Elimination

Low (40) suggested, considering the “idiopathic” cause of some diseases that “this unfortunately fosters the attitude that behavioral disorders are only of secondary importance to “medical” disorders,” “a behavioral diagnosis should be an active process rather than occurring passively after ruling out all other “more legitimate” possibilities.” Hence, behavioral ulcerative dermatitis should not now be a diagnosis by eviction but a positive diagnosis with association of both negative (no somatic cause) and positive criteria (clinical characteristics, association with poor welfare score). The clinical characteristics are pruritus without primary skin lesions and localization of lesions in the area of grooming by scratching. Optional criteria like a chronological relationship of the occurrence of pruritus with one or several life events that could have psychological repercussions are rarely or never reported by the owner.

Clinical signs are self-induced lesions (excoriations, ulcers erosions, scars, alopecia) localized in the areas of grooming by scratching. Skin lesions, in our study, can be single or multicentric, with an asymmetric or symmetric distribution. The most frequent localizations were the neck, the temporal area of the head, and the shoulder. They were localized to one region (2/3 of cases in our study) or had several localizations (1/3 of cases in our study). Median age of the onset of scratching (19 months in our study) was similar to previous publications (36).

Differential diagnoses have to be made with a dermatological disease. Signs orienting toward a dermatologic disease are the presence of cutaneous lesions, which the cat is unable to induce itself (i.e., a miliary dermatitis, pustules, scales, exfoliative dermatitis, linear eosinophilic granuloma, eosinophilic plaques, urticaria…). However, a pruriginous dermatologic affection like severe atopic dermatitis may be accompanied by self-induced wounds. In this case, the dermatologic examination shows a mixture of inflammatory lesions with a characteristic topography and excoriations (41).

It is well known in human that psychosomatic factors frequently enhance somatic sensations like pruritus or pain (42). Some humans have only a somatic disease, others have a specific psychogenic pruritus, but the broad majority of patients with pruritus suffer from somatic disease and symptoms are modulated by psychosomatics factors (32). These knowledges are relevant in the cat (Cochet-Faivre, Personal Observation.), for this reason, a meticulous dermatologic examination is required.

### Pathogenesis

It is described in human that sensory, motor, and affective areas are activated at the same time when pruritus occurs (32, 43–46). The very important role of brain in the pathogenesis of pruritus confirms that a specific psychogenic pruritus is possible in human (32, 47).

The release of peripheral inflammatory mediators by scratching sensitizes pruriceptors (peripheral sensitization), while this chronic skin inflammation facilitates spinal and central itch processing, resulting in touch-evoked pruritus (central sensitization). The existence of central sensitization for itch improves our understanding of psychogenic pruritus and of the transitory efficiency of antipruritic treatment (32). In cats, the same process has to be proved but is suspected. Transitory efficiency of corticosteroids, observed in the majority of cats recruited, could be explained by its action on the peripheral and central inflammation. Besides, in humans, brain inflammation is described with obsessive compulsive disorder (48).

## Conclusion

To our knowledge, this study is the first to consider IUD as a behavioral disease and as an indicator of poor welfare. We hence propose to rename IUD to “behavioral” ulcerative dermatitis (or self-induced ulcerative dermatitis), following the presence of these three items; (1) all cutaneous lesions are self-induced lesions leading to self-harm in the area of grooming by scratching, (2) this repetitive behavior is associated with a poor welfare score, (3) complete healing follows environmental change to match specific ethological cat’s needs.

Further studies involving a wider sample of cats could help to better define this behavioral disorder, linked to a mal-adaptation of the cat to its environment. The welfare scoring could also be a useful tool to investigate other repetitive behaviors such as tail chasing or self-induced alopecia.

## Ethics Statement

Cats were treated according to the CHUVA ethics, since they were recruited as patients from dermatology consultations or vaccine consultations for control cats. Ill cats were under care at the CHUVA, and owners of control cats were asked to fill out a questionnaire. All owners gave their written consent to participate to the study.

## Author Contributions

ET and NC-F conceived the presented idea. AB, NC-F, and ET have recruited the cases. CG developed the welfare score. All authors drafted read and approved the final manuscript.

## Conflict of Interest Statement

The authors declare that the research was conducted in the absence of any commercial or financial relationships that could be construed as a potential conflict of interest.
